# Secure and Efficient High Throughput Medium Access Control for Vehicular Ad-Hoc Network

**DOI:** 10.3390/s21144935

**Published:** 2021-07-20

**Authors:** Mohammed Abdulhakim Al-Absi, Ahmed Abdulhakim Al-Absi, Rui Fu, Ki-Hwan Kim, Young-Sil Lee, Byung-Gook Lee, Sang-Gon Lee, Hoon-Jae Lee

**Affiliations:** 1 Department of Computer Engineering, Graduate School, Dongseo University, Busan 47011, Korea; d0185123@kowon.dongseo.ac.kr; 2 Department of Smart Computing, Kyungdong University, Gosung 24764, Korea; 3 Blockchain Laboratory of Agricultural Vegetables, Weifang University of Science and Technology, Weifang 262700, China; furui19891209@wfust.edu.cn; 4 International College, Dongseo University, Busan 47011, Korea; ghksdl90@naver.com (K.-H.K.); youngsil.lee0113@gmail.com (Y.-S.L.); 5 Division of Information and Communication Engineering, Dongseo University, Busan 47011, Korea; lbg@dongseo.ac.kr (B.-G.L.); nok60@gdsu.dongseo.ac.kr (S.-G.L.)

**Keywords:** V2V, authentication, security, DSRC, privacy, MAC

## Abstract

The evolution of the internet has led to the growth of smart application requirements on the go in the vehicular ad hoc network (VANET). VANET enables vehicles to communicate smartly among themselves wirelessly. Increasing usage of wireless technology induces many security vulnerabilities. Therefore, effective security and authentication mechanism is needed to prevent an intruder. However, authentication may breach user privacy such as location or identity. Cryptography-based approach aids in preserving the privacy of the user. However, the existing security models incur communication and key management overhead since they are designed considering a third-party server. To overcome the research issue, this work presents an efficient security model namely secure performance enriched channel allocation (S−PECA) by using commutative RSA. This work further presents the commutative property of the proposed security scheme. Experiments conducted to evaluate the performance of the proposed S−PECA over state-of-the-art models show significant improvement. The outcome shows that S−PECA minimizes collision and maximizes system throughput considering different radio propagation environments.

## 1. Introduction

**Figure 1:**
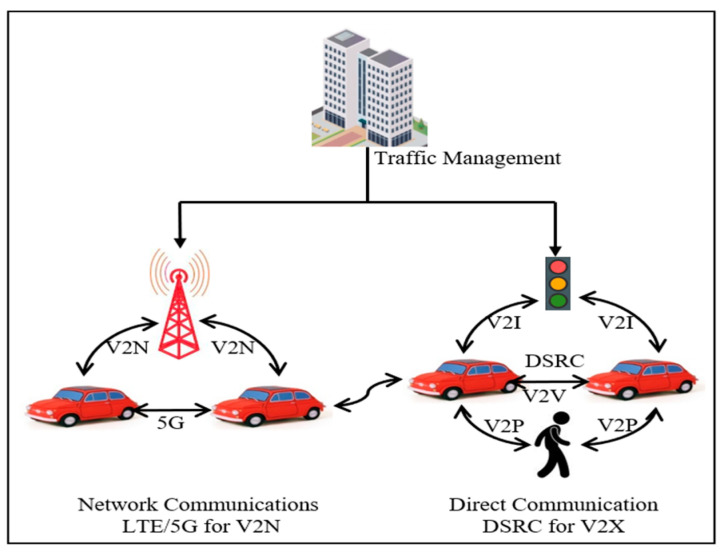
The architecture of vehicular ad hoc communication.

VANET is a special type of Mobile ad hoc Network (MANET) where vehicles/devices act mobile devices, and their mobility is defined by network road topologies [1]. The goal of VANET is to assist drivers and subscribers with a reliable and safe atmosphere. The communication in VANET takes place from Vehicle to Infrastructure (V2I), Vehicle to Vehicle (V2V), and Vehicle to Everything (V2X) which is a combination of both. Each vehicle is equipped with sensors such as onboard unit (OBU), Bluetooth, 3G/4G/5G, and Wi-Fi that has communication and computational capabilities [2] (Table 1). Roadside unit (RSU) with dedicated short-range communication (DSRC) [3,4] is the public infrastructure that is fixed on the roadside to provide internet to the vehicle [5,6]. A typical VANET communication is shown in Figure 1. DSRC is a dedicated short-range communication, which is a one-way or two-way short-range to medium-range wireless communication technology based on the IEEE802.11p protocol [7,8]. In October 1999, the United States Federal Communications Commission (FCC) allocated 75 MHz of spectrum in the 5.9 GHz frequency band for use by Intelligent Transportation Systems (ITS) [9], which is now one of the two technologies that implement V2X [10].

**Figure 2:**
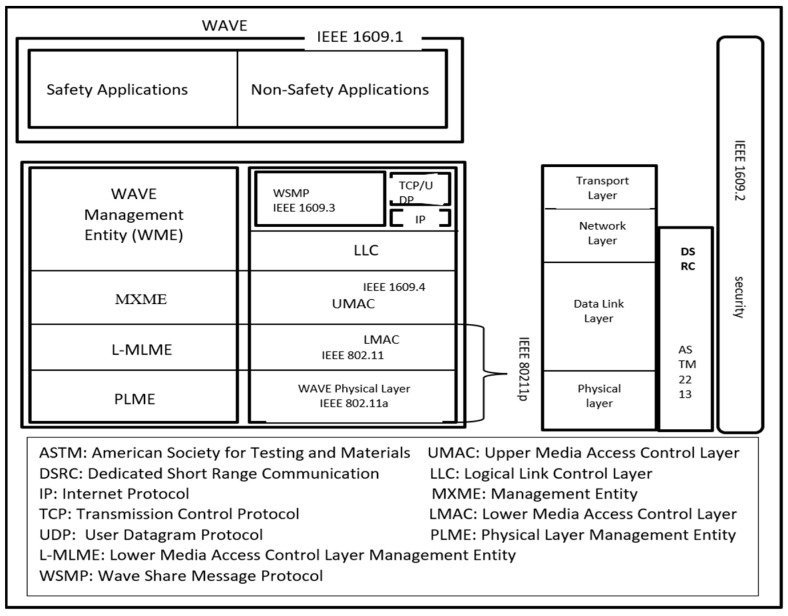
WAVE model.

DSRC adopts the IEEE 802.11p standard specification for wireless communication [11], where each device broadcast a safety-related message every 100–300 milliseconds, which possess vehicle driving-related data, such as speed, location, and driving status (e.g., waiting for the signal, regular driving, traffic jam, etc.), to neighboring devices. With the acquired information, other vehicles can make a timely decision in cases such as traffic jams, emergent braking, and accidents. As mentioned in Figure 2, works at the media access control and physical layers act strictly, and it is worth noting that IEEE 802.11p is limited by the scope of IEEE 802.11. The operational functions and complexity of DSRC are taken care of by upper layers of IEEE 1609 standards. Based upon management activities defined in IEEE P1609.1, the security protocols defined in IEEE P1609.2 and the network-layer protocol defined in IEEE P1609.3, the applications utilized in the WAVE environment are depicted by these standards. Compared to 802.11p, IEEE 1609.4 is higher in level, and the operation of higher layers without the necessity the physical channel access parameters is supported [12].

The DSRC ecosystem has implemented various functions and fully tested V2X applications for more than ten years [13]. However, DSRC provides a complete set of interoperable solutions [14,15]. The key advantage of DSRC is that it can “see the surrounding corners” without other sensors. DSRC technology with high mobility can handle rapidly changing environments at a speed of up to 500 km/h even if an obstacle is suddenly detected, and its range exceeds 1 km [16,17]. DSRC makes it possible for users of the road to be connected, which guarantees the reliability of V2V and V2I. The European Commission believes that the use of this technology is expected to reduce the probability of local motor vehicle accidents to zero in 2050 [18].

C−V2X is a wireless communication technology for cellular vehicles. At present, the market is upgrading to 4GC−V2X, and 5GC−V2X is in the process of standardization [19]. In the future, it will arrive at the same time as the implementation of intelligent vehicle interconnection. C−V2X is supported by many mobile operators, major mobile device manufacturers, and automakers, including Audi, BMW, Daimler, Ford, Tesla, and Toyota. Mobile operators, equipment suppliers, and vehicle manufacturers are joining forces to test C−V2X [20,21].

Though VANET offers tremendous benefits, at the same time, Internet of Vehicles (IoVs) are prone to security attacks. As a result, the security issues must be addressed before practical usage [22]. Therefore, recently, extensive research on network security protocols has been carried considering characteristics such as highly dynamic and self-organizing network topology which is applied to IoV. Among them, identity authentication methodology is an effective way to provide data security [23]. Similarly, authentication and key management techniques have been widely studied and applied extensively in other fields such as the Internet of things (IoT), smart grids, mobile cloud computing, etc. [24,25,26]. The desired security on such a platform must first guarantee message integrity. Secondly, to prevent impersonation attacks, the data sender must be authenticated. In addition, user privacy [7,27] concerns must be taken care of, where the position, identity, and movement of a particular user must not be accessible to any third party. However, in VANET, it is not desired to have such an unconditional privacy-preserving scheme. Since the malicious/intruder vehicles must be tracked and punished in case of any malicious activity carried out.

The authentication scheme can be broadly classified into the following three categories: cryptography-based [28,29], trust-based [30,31], and hardware-based [32,33]. In the V2V semi-trust model, organizations involved in protecting privacy are not well suited to deliver high-throughput security applications and smart entertainment applications. To protect the confidentiality of user information, many studies on the state of the art mainly use encryption technology. The encryption technology is mainly based on symmetric or asymmetric encryption keys and decryption. We review the use of secure servers or third-party servers as traditional methods of computing and distributing the key to an authorized organization [34].

The authorities involved in preserving privacy in V2V semi-honest trust model are not suitable for provisioning high throughput safety and smart infotainment application. To preserve the privacy of user data, in literature, many researchers have predominantly adopted cryptography techniques. The cryptography technique relies on the keys for encryption and decryptions, where keys are symmetric or asymmetric. To compute keys and distribute among authorities, a secure or third-party server is considered [35,36]. The usage of third-party servers incurs the overhead of key computation, storage, and distribution, also known as the initialization phase. Post completion of the initialization phase, the message is secured using cryptography and is shared among vehicles. The design of the proposed Secure Performance Enriched Channel Allocation (S−PECA) model aims to eliminate the need for the local message available with the authorities to be released for provisioning high throughput safety and infotainment application. Firstly, we develop an efficient MAC namely PECA [37] that overcomes the NP−hard problem [38] of channel sharing in ENCCMA [39] MAC design. Secondly, a security model is designed using commutative RSA, namely, S−PECA.

The contributions of this research paper are:

- RSA cryptography technique with commutative key helps maintain message integrity and privacy.
- Our proposed scheme minimizes the computational overheads associated with preserving the privacy of the S−PECA model (namely key computation, exchange, and distribution using external entities).
- The S−PECA model preserves or protects the privacy information in the presence of untrusted or dishonest authorities.
- Compared with the existing design, the provision of our design has a much lower security overhead.
- The result obtained shows that the suggested design minimizes collision and maximizes system throughput.

The remainder of the work is as follows. In Section 2, the literature review is carried out. The proposed channel allocation model is discussed in Section 3. In the penultimate section, experiments and simulations are presented. The last section provided and discussed the conclusion with future work.

## 2. Literature Review

A comprehensive survey of the existing security design is carried out for provisioning security to VANET in this section. In [35] presented an efficient pseudonymous authentication design to protect user personal information. They presented multiple hierarchies of pseudonyms based on user sessions. A session with smaller timestamp pseudonyms is used for communication among semi-trusted authorities and longer session timestamp pseudonyms are used for communication among vehicles. Their model overcomes the storage and computation overhead of certificate revocation lists and group-based approaches. Experimental outcomes show it minimizes end-to-end package delay and delivery ratio. However, they consider only honest but curious server and suffer from trust-related issues concerning certificate authority. The study in [40] proposes a geo-routing protocol for the introduction of the Location Errors Record (LER-GR), evaluating the position error of neighboring vehicle compounds using the error calculation method according to the Rayleigh distribution and development of position prediction and correction technology based on Kalman filter and prediction of the position of neighboring vehicles. The authors in [41] use the reconfigurable intelligent surface (RIS) to enhance the Physical Layer Security (PLS) in VANET. However, they have presented two network system models: the first is vehicle-to-vehicle communication through RIS and source-based access points, and the second is information of VANET with RIS based relay deployed in the building as mentioned in [42]; the signature verification takes around 20 ms by the onboard unit at a 400 MHz processor. This might not be a problem in sparse areas, but in dense areas, it could cause significant delays in the message verification process. The Certificate Revocation List (CRL) is another limitation of the pseudonym methods, i.e., certification authority creates a set of public vehicle key certificates. Then, the vehicle uses the private key to sign the beacon and broadcasts the signal using the corresponding public key certificate. However, in the case of revocation, you need to add all certificates of revoked users to the CRL. Hybrid Intrusion Detection System (D2H-IDS) is used to separate trusted service requests from invalid requests that were created during malicious attacks used to prevent security attacks [43]. Reference [44] designed an approach to optimize scheduling, routing, and access control while reducing network congestion, securing a slot allocation for reserved traffic, securing network reliability, and maximizing approval of network flows. Reference [45] presented a reliable and secure connection to reduce unnecessary communication between edges by relying on the transport protocol between nodes in smart cities. Reference [46] proposed HIBS-K Sharing, given different types of communication devices, which is suggested to share a hierarchical identifier-based signature key for Automotive vehicles. Similarly, the authors in [47] presented a hardware-based security design to provide security to address the trust-related issue of [35]. They considered a hybrid security model and presented a design to preserve the privacy of vehicular communication. Their model considers dual authentication based on different IoV scenarios. Firstly, the onboard unit computes a temporary encryption key and anonymous identity to initialize the authentication session. Then, the trusted authority can evaluate the authorized vehicles’ anonymous and real identities. The vehicle reputation is evaluated based on past transmission based on which a session key can be established. Their model preserves privacy and minimizes key exchange overhead. Nonetheless, the tamper-proof device may not guarantee all the VANET security requirements [36] and incurs communication overhead. To address this, ref. [36] presented a secure privacy-preserving authentication scheme. Their model does not rely on any hardware and attained a much higher data rate than the batch verification scheme by using the binary search method and cuckoo filter. They have achieved a great improvement in performance over the state-of-the-art technique. Since it is paired for free, the mapping point segmentation function is not used. An extensive survey carried shows the cryptography approach plays a significant in preserving the user and adopting third-party servers and public-key cryptography incurs communication and key management overheads. To address research challenges, in the next section, we present a secure MAC design using commutative RSA.

## 3. Secure VANET Communication (SVC) Using Commutative RSA Technique

This work presents a secure MAC protocol design for VANET. Firstly, we present a Perform Enriched Channel Algorithm (PECA) for the shared channel and the non-shared channel in VANET. First, we choose the best channel available to the user according to the throughput gain requirements. The users do not share channels here; the user enters the channel during a specified period and leaves the channel so that other users can access it. However, this algorithm cannot use the bandwidth effectively. This is because the channels are not shared. To solve this problem, the second algorithm proposes a shared channel allocation algorithm. Here, a group of users shares channels between neighboring users. This algorithm utilizes bandwidth efficiently, which aids in minimizing collision and maximize system throughput Then we present a CRSA based security design S−PECA for secure communication among vehicles. The list of notations and symbols used in this paper is given in Table 2.

(a) Non-shared channel allocation (NSCA):

Where $T_{x}$ defines the channels assigned to vehicle/node x ($T_{x}\;\cap \;T_{y}=\emptyset$, $x\neq y);\;l_{x,y}$ is the likelihood that channel y is accessible at vehicle x. The mechanism of the non-channel shared allocation algorithm (if each participant is given a channel to transmit in a specified time, it is called Non- Shared Channel Algorithm) allows the vehicle to be allocated channels repeatedly to maximize throughput. However, in each channel frequency, each x vehicle will calculate the throughput gain when assigning the best channel under the following condition

(1) $$y_{x}^{\prime }=\underset{y\in T_{z}}{argmax}l_{x,y},$$

This throughput Gain is calculated as

(2) $$\delta C_{x}=C_{x}^{z}-C_{x}^{q}=\left[1-\left(1-l_{xy_{x}^{\prime }}\right)\underset{y\in T_{x}}{\prod }\left(1-l_{xy}\right)\right]-\left[1-\underset{y\in T_{x}}{\prod }\left(1-l_{xy}\right)\right]=l_{xy_{x}^{\prime }}\underset{y\in T_{x}}{\prod }\left(1-l_{xy}\right)$$

**Figure 3:**
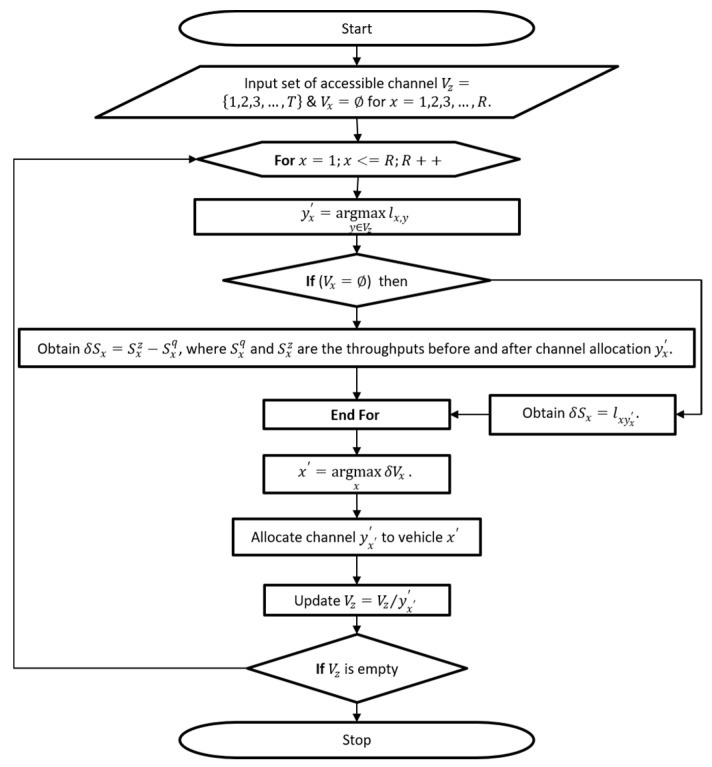
Flow diagram of proposed non-shared channel allocation.

It can be noticed from Equation (2), $\delta C_{x}$ is reduced with each repetition of the assignment, where $C_{x}^{z}$ and $C_{x}^{q}$ are the throughputs before and after channel, T is the total number of channels in the network, and if $T_{x}$ increases, then $\underset{y\in T_{x}}{\prod }\left(1-l_{xy}\right)$ tends to zero. However, given this situation, the recommended NSCA is defined in flow diagram 1 as shown in Figure 3. First, we initialize the set available channel for all vehicles, then for all vehicles do allocate the best available channel to the vehicle (a channel with maximum likelihood). Then, check if the set of channels assigned to the vehicle is not equal to zero. If it is not equal to zero, then, obtain throughput gain before and after channel allocation. If it is equal to zero, then, the likelihood of throughput gain is assigned. Assign each vehicle maximum throughput, and then, allocate channel with maximum throughput to vehicles. Update the allocated channel information with maximum throughout to each vehicle. If the allocated channel is empty, then, terminate, or else go to step 2.

Note: we run flow diagram 1, to get channels set assigned to each device/vehicle, and according to these channels, Equation (5) can be utilized to calculate the throughput. Therefore, this work’s goal is to achieve maximum throughput in the network to obtain channel allocation performance. However, we consider the throughput gained by x vehicle/device where $C_{x}$ and $e_{xy}$ represent the channel allocation decision. However, if the y channel is set for x vehicle, $e_{xy}$ is set 1, else $e_{xy}$ is set to 0. However, the gain throughput issue is shown as follows:

(3) $$\underset{E}{max}\overset{R}{\underset{x}{\sum }}C_{x}.$$

We have the following commitment to allocate the non-shared channel as

(4) $$\overset{R}{\underset{x}{\sum }}e_{xy}=1\;\forall y$$

Now, we can calculate the throughput gained by x vehicle on non-shared channel assignment according to the following formula: $T_{x}$ is the x vehicle/device assigned channel group, and $l_{xy}$ is x vehicle channel y accessibility. However, $C_{x}$ is calculated as follows:

(5) $$C_{x}=1-\underset{y\in T_{x}}{\prod }l_{xy}^{\prime }=1-\overset{K}{\underset{y=1}{\prod }}{\left(l_{xy}^{\prime }\right)}^{e_{xy}}$$

where $l_{xy}^{\prime }=1-l_{xy}$ is the probability of x vehicle/device not accessing the y channel, and $1-\underset{y\in T_{x}}{\prod }l_{xy}^{\prime }$ is the probability of x vehicle accessing the channel. However, each vehicle can use one channel utmost, so the highest throughput is 1. The bound in Equation (4) is not required in the channel assignment technique. Moreover, solving Equations (3) and (4) are NP−hard problems because this is a nonlinear integer program.

(b) Shared channel allocation (SCA):

A shared channel (If the channel is shared between neighboring vehicles, then each vehicle has a specific time to do the transmission. However, the time required to reach the channel is determined by two factors, maximum throughput, and reduced collision for multi-user vehicle grid in the duct help improve throughput performance. However, they create MAC overhead due to multi-user allocation access channel conflict. Therefore, an optimized channel allocation method is needed to overhead for redundant design and balance throughput.

**Figure 4:**
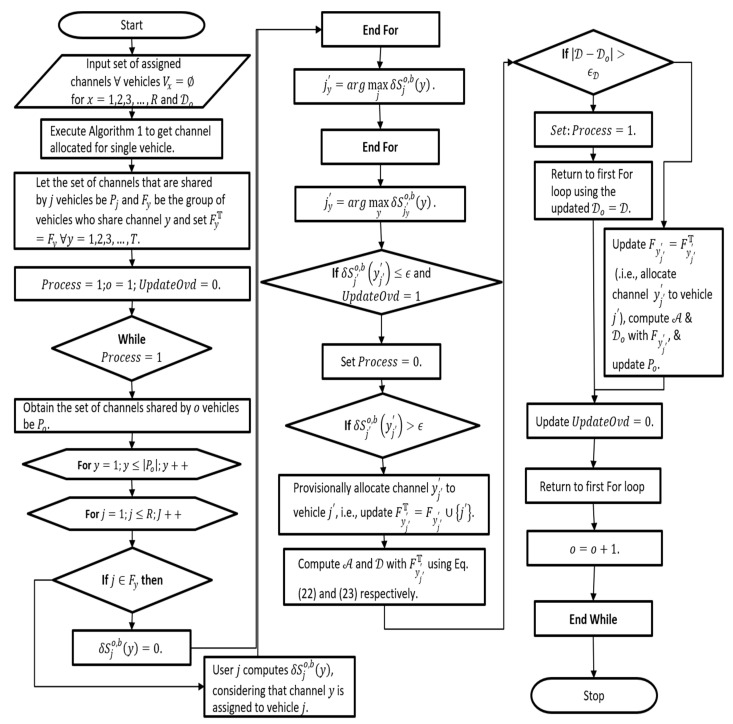
Flow diagram of proposed channel shared allocation.

The channel allocation model includes two steps. In the first as shown in Figure 2, single-vehicle channel assignment information is computed using flowchart 1. The following deals with multi-user channel allocation by assigning channels assigned to specific vehicles to other vehicles. Here, we model the SCA algorithm as shown in flow chart 2 in Figure 4.

First, assigned accessible channels for all vehicles. Execute algorithm to obtain channel assigned to single vehicle then consider a group of channels which are shared by set vehicles/device and set of vehicles that shares channel among vehicles in the network. Update overhead to zero and set process to 1. Initialize the while loop and the options set of channels shared by vehicles. Initialize for loop for all shared vehicles in the network, and then, find if a vehicle belongs to the vehicle that shares their channel. If the channel is shared, then the assigned estimated throughput gain of the channel is zero; else, the vehicle computes throughout the gain considering the channel is allocated to vehicles. Assign estimated throughput gain for channel to the vehicle, and again, estimate throughput gain for the channel to the vehicle that shared the channel. If the estimated throughput gain is less than or equal to collision likelihood and overhead tradeoff, update overhead to 1 and set process to zero, and then, end the while. However, if the estimated throughput gain is greater than the collision likelihood, then provisionally allocates a channel to a vehicle that shares the available channel. Compute contention window and MAC overhead for the vehicle that shares the available channel. If the current MAC overhead is (minus initialized MAC overhead) greater than collision likelihood then, process overhead is set to 1. (Means it incurs MAC overhead as a result new channel need to be identified). Initialize the loop for all shared vehicles using updated MAC overhead. If the current MAC overhead is not greater than collision, then assign a channel to the shared vehicle. Compute MAC overhead and contention window and update group of channels shared by a set of vehicles. Update overhead to zero (no overhead is incurred in channel allocation of the shared channel) and increment the number of vehicles using the shared channel.

However, the calculation of indicators is a very difficult task. Therefore, by taking MAC overhead D<1, we calculate the channel allocation throughput gain. (D represents MAC overhead incurred in allocating a set of channels to the vehicle. The likelihood of collision due to the overhead incurred in the MAC layer due to channel allocation will be a range of 0 to 1.) Note: D overhead is based on the output of the channel assignment. The calculation of D is later described in the subsection of this paper.

Consider y channel is the channel shared in $x_{1},x_{2},\ldots ,x_{T}\;\mathrm{the}\;\mathrm{vehicle},$ and T are the sharing vehicles of y channel. Here, if the y channel is assigned to a particular x vehicle, then we compute the gain throughput of that vehicle. If other vehicles $x_{1},\;x_{2},\ldots ,x_{T}$ do not use this y channel or are unreachable to the y channel, x vehicle can use the y channel which can increase throughput gain transmission when taken into consideration. The throughput gains of x vehicle and y channel are calculated as follows:

(6) $$\delta C_{x}^{T,b}\left(y\right)=\left(1-\frac{1}{T}\right)\left(1-D\right)l_{xy}\left(\underset{o\in T_{x}}{\prod }{\bar{l}}_{xo}\right)*\left(1-\underset{o\in T_{*}^{s}}{\prod }{\bar{l}}_{xo}\right)\overset{T}{\underset{n=1}{\sum }}\left[{\bar{l}}_{x_{n}y}\left(\overset{T}{\underset{m=1,m\neq n}{\prod }}l_{x_{m}y}\right)\right]$$

where b is the estimated throughput gain of user and channel; ${\bar{l}}_{xo}$ is the number of users sharing the channel; $1-\underset{o\in T_{*}^{s}}{\prod }{\bar{l}}_{xo}$ is the likelihood of the commonly shared channel users. s is the common shared channel; n is the shared channel user number; m is the user’s number uses the shared channel; $\overset{T}{\underset{m=1,m\neq n}{\prod }}l_{x_{m}y}$ is the likelihood computation of throughput gain on a shared user channel.

The S−ENCCMA model is implemented using the RSA with the commutative key mechanism. S−ENCCMA uses the ENCCMA real-time communication MAC protocol [37]. However, to provide access to real-time, ENCCMA combines Cognitive Radio, TDMA, and FDMA techniques. The ENCCMA MAC protocol can block signal transmission, and this aids to improve system efficiency. Therefore, ENCCMA does not consider user privacy, nor does it provide message authentication security. The proposed security model is presented in the next subsection.

**Figure 5:**
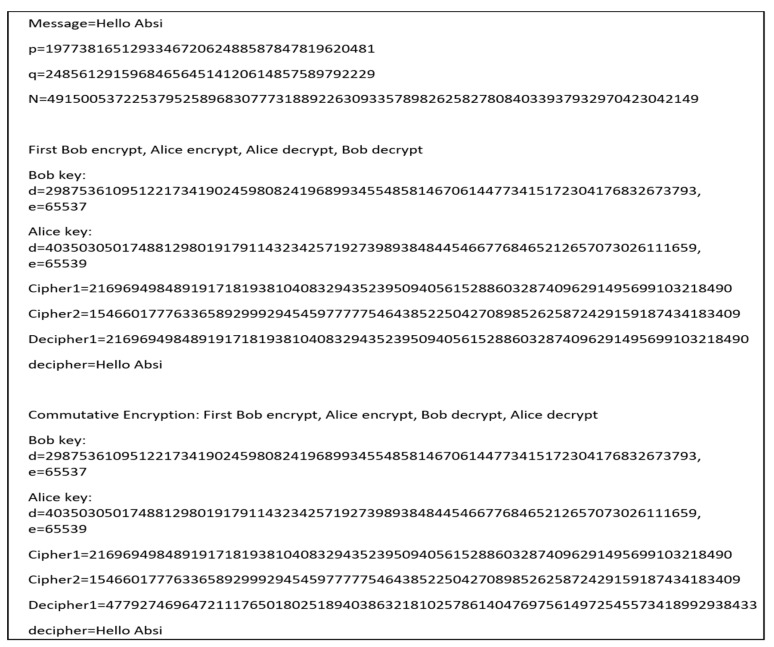
Encryption in the correct order and then in the incorrect order.

(c) RSA with commutative key:

Generally, with encrypting, we first encrypt with Bob’s key, then encrypt with Alice’s key, and then decrypt with Alice’s key and Bob’s key. The exchange cipher allows decoding in any order. An important factor is that Bob and Alice need to share the values of p and q. For example, Figure 5 shows the encryption in the correct order and then in the incorrect order with the use of Prime size 128 (bits).

However, we propose a safe and effective CRSA algorithm for data authentication between participant’s vehicles/devices in a V2V environment. To enable safe data communication among the corresponding vehicle in the V2V environment, it is a noble commutative RSA method which indicates that in order encryption can be performed in the same manner without affecting the results of encryption and decryption technique.

A secure communication model can be realized only when message transmitted over the communication channel is protected and cannot be collided. To achieve this, cryptography mechanisms are generally considered. Therefore, the S−PECA proposed here adopts CRSA algorithm. The S−PECA considers two prime param $A_{a}^{C}$ and $B_{b}^{C}$ initialized amongst all the vehicles of the region. Let ${ℛ}_{X}$ and ${ℛ}_{Y}$ represent the region member required to securely communicate over the secure channel. To compute the encryption keys and decryptions key pairs of the CRSA algorithm, the property $L^{C}$ and $M^{C}$ are evaluated using the following:

(7) $$L^{C}=\left[\left(A_{a}^{C}\right)\times \left(B_{b}^{C}\right)\right]$$

(8) $$M^{C}=\left[\left(A_{a}^{C}-1\right)\times \left(B_{b}^{C}-1\right)\right]$$

From the above expression, it can be seen that $L_{X}^{C}=L_{Y}^{C}$ and $M_{X}^{C}=M_{Y}^{C}$ for X and Y. The key pair for encryption of X and Y are signified as follows:

(9) $$\left(L_{X}^{C},{ℰ}_{X}^{C}\right)\;\mathrm{and}\;\left(L_{Y}^{C},{ℰ}_{Y}^{C}\right)$$

The parameter ${ℰ}^{C}$ has obtained by arbitrarily selecting the parameter like it is a co-prime of $M^{C}$, in another expression

(10) $$F_{G}\left({ℰ}^{C},M^{C}\right)=1$$

where $F_{G}\left(u,v\right)$ denotes the largest common factor function between u and v.

The decryption pair key of X and Y is described by $\left(L_{X}^{C},D_{X}^{C}\right)$ and $\left(L_{Y}^{C},D_{Y}^{C}\right)$. The $D^{C}$property is evaluated as follows:

(11) $$D^{C}={\left({ℰ}^{C}\;\right)}^{-1}\left|L^{C}\right|\;$$

Let $E_{U}$ indicate the encrypted U message. The encryption process is as follows

(12) $$E_{U}=V^{{ℰ}^{c}}\left|L^{c}\right|$$

The CRSA decryption process is expressed on Y encrypted message as

(13) $$D_{V}=V^{D^{c}}\left|L^{c}\right|$$

(d) Proof of commutative RSA model:

If the U message is encrypted with X and then encrypted with Y, the commutative RSA can be demonstrated by the SVC model. As for the encryption performed with Y, if it is encrypted at X, the message result is the same and can be expressed as follows:

(14) $$E^{Y}\left(E_{U}^{X}\right)\equiv E^{X}\left(E_{U}^{Y}\right)$$

(15) $$E^{Y}\left(U^{{ℰ}_{X}^{C}}\left|L_{X}^{C}\right|\right)\equiv E^{X}\left(U^{{ℰ}_{Y}^{C}}\left|L_{Y}^{C}\right|\right)$$

(16) $$U^{\left({ℰ}_{X}^{C}\times {ℰ}_{Y}^{C}\right)}\left|L_{X}^{C}\right|=U^{\left({ℰ}_{Y}^{C}\times {ℰ}_{X}^{C}\right)}\left|L_{Y}^{C}\right|$$

As $L_{X}^{C}=L_{Y}^{C},$ it can be said that

(17) $$U^{\left({ℰ}_{X}^{C}\times {ℰ}_{Y}^{C}\right)}\left|L_{X}^{C}\right|=U^{\left({ℰ}_{Y}^{C}\times {ℰ}_{X}^{C}\right)}\left|L_{X}^{C}\right|$$

and therefore,

(18) $$E^{Y}\left(E_{U}^{X}\right)\equiv E^{X}\left(E_{U}^{Y}\right)$$

**Figure 6:**
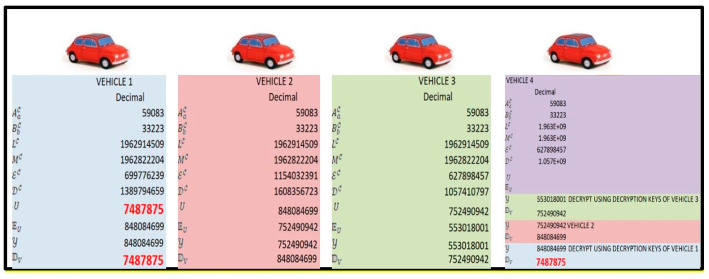
Example for CRSA.

Each vehicle computes its public and private key using the proposed commutative RSA algorithm. Hop-based communication is adopted for data transmission among vehicles. Each vehicle encrypts the data using its own public key. The receiver performs decryption operations based on the number of times it is encrypted using its commutative keys of participating vehicles. The proposed model preserves data and user’s privacy, and an intruder can be tracked using the user’s commutative keys. First, once established the key management, the key management center will distribute two prime numbers A and B to all VANETs which are the same. Then, it will calculate L and M at each VANET node. Based on these two, each vehicular node will compute the encryption and decryption keys. Second, once established the key exchange and once all the vehicles do their encryption and decryption keys, they will inform the key management that it is over. For example (Figure 6):

1. Key setup:
  (a) The same values of *A* and *B* are considered in all VANETs distributed by the key management center.
  (b) *L* and *M* are calculated at each VANET node.
  (c) Using random number generator encryption parameter E and decryption parameters D.
2. Key exchange:
  (a) Vehicle 1 is the source, and vehicle 4 is the destination.
  (b) Vehicle 4 will get decryption keys of vehicle 1, 2, and 3 (Vehicle 1 (1962914509,1389794659), Vehicle 2 (1962914509,1608356723), Vehicle 3 (1962914509,1057410797)).
3. Secure data exchange (no original data are exposed/revealed):
  (a) Vehicle 1 will encrypt the data and send them to 2.
  (b) Vehicle 2 will encrypt data and send them to 3.
  (c) Vehicle 3 will encrypt the data and send them to 4.
  (d) Vehicle 4 will decrypt the data using keys of vehicles 3, 2, and 1 to get the original data.

In the normal RSA or Elliptical Curve Cryptography (ECC) [48], when you encrypt encrypted data again, data get corrupted. Therefore, on decryption, the data cannot be recovered. Therefore, in our mechanism, the user does not need to decrypt the data; the user can just encrypt using his key and forward it. The normal RSA implementation might not be very fruitful, and it remains unexplored even with recent and optimized encryption techniques. Hence, approaches like commutative characteristics, which means that the order in which encryption takes place does not affect the decryption process if it is done in the same way and avoids security breaching, can be implemented. The unique characteristic of commutative RSA cryptosystem is that it can facilitate the reorder decryption which is unique and effective itself. On the other hand, in most existing approaches, the public key cryptosystems employ a key exchange approach that ultimately causes the increase in computational overheads for key exchange, and alternatively, in individual transceiver, the encryption and decryption are a must, and thus somewhere, the efficiency as well as security would be compromised. Therefore, the consideration of commutative RSA (CRSA) might be an optimum solution for accomplishing an efficient and most secure communication for multi-channel V2V vehicular ad hoc smart infotainment applications.

(e) Computation of contention window:

To reduce the overhead probability between contending V vehicles considering security provisioning, contention window A is computed (example of contention window: a vehicle that wants to transmit a packet must first request a channel) [37]. Indeed, there is a tradeoff between collision probability and overhead of MAC protocol that is influenced by A (i.e., decreasing the A value increases the probability of the collision, in the cost of MAC lower overhead (lower MAC overhead: overhead incurred in defining contention window size)) and vice versa. However, each vehicle chooses some equal back-off time. However, the higher the probability of a collision, the higher the probability of a first collision because the number of vehicles involved decreases. (For example, firstly, 10 vehicles contend, and out of those, 5 get contention, so the collision likelihood is higher (20%). Then, only the remaining 5 vehicles contend for the channel. Therefore, the likelihood of collision comes down.)

Let ${ℒ}_{u}$ be the probability of the first collision. Consider constraint ${ℒ}_{u}\leq \epsilon L$, where ϵL is the tradeoff between collision probability and management overhead to determine A contention window. For r vehicles in the window contention stage, ${ℒ}_{u}$ is evaluated as a function of A. If there is no loss, consider the arbitrary back-off time of r vehicles (arbitrary back-off time of r vehicles is the random time selected by a set of r vehicles for contention (that is, r number of vehicles waiting for a while for channel access after detection of the first collision)) are arranged as $g_{1}\leq g_{2}\leq \cdots \leq g_{r}$. ($g_{r}$ is the random backoff time of each participating user in channel contention; r is the participation number of vehicles in the network. The first vehicle has the least waiting time, and the last vehicle has the maximum waiting time). Suppose r vehicles/devices in the contention stage; the probability of the 1st collision is shown as follows:

(19) ℒu(r)=∑y=2rL(y vehicles/device collide)=∑y=2r∑x=0A−2Ury(1A)y(A−x−1A)r−y

Each component in the double heaps shows the probability of a collision if y vehicles choose the same correction value for x. However, the probability of the first collision is computed as follows:

(20) $${ℒ}_{u}=\overset{R}{\underset{r=2}{\sum }}{ℒ}_{u}^{\left(r\right)}*L\left\{r\;vehicle/device\;contend\right\}$$

where L{r vehicle/device contend} are the possibility of participating in the vehicles r in the contention stage and the Equation (19) is used to calculate ${ℒ}_{u}^{\left(r\right)}$. To rate$\;{ℒ}_{u}$, we derive L{r vehicle/device contend}. If we have access to one channel $T_{x}$ and all channels $T_{x}^{S}$occupied, we can prove that x vehicle will participate in the contention. The probabilities of this scenario are expressed as:

(21) $${ℒ}_{S}^{\left(x\right)}=L\left\{\begin{array}{c} 1\;channel\;at\;T_{x}^{s}\;is\;accessible\;and\\ \;all\;channels\;at\;T_{x}\;occupied \end{array}\right\}=\left(\underset{y\epsilon T_{x}}{\prod }{\bar{l}}_{xy}\right)\left(1-\underset{y\epsilon T_{x}^{s}}{\prod }{\bar{l}}_{xy}\right)$$

The probability of the scenario in which the r vehicles users in the contention phase (contention phase is the period of the request of a channel for data transmission) is

(22) $$L\left\{\;vehicle/device\;r\;contend\right\}=\overset{U_{R}^{r}}{\underset{k=1}{\sum }}\underset{x\in \wedge_{k}}{\prod }{ℒ}_{S}^{\left(x\right)}\underset{x\in \wedge_{R}\backslash \wedge_{t}}{\prod }{ℒ}_{S}^{\left(x\right)}$$

$\wedge_{R}$ is the group of all vehicles R ({1,2,…;R}), $\wedge_{k}$ is a particular group of r users. Output substitution of Equation (22) into Equation (20); ${ℒ}_{u}$ can be calculated. Nevertheless, it becomes possible to define A as

(23) $$A=\min\left\{A|{ℒ}_{u}\left(A\right)\leq \epsilon L\right\}$$

where, ${ℒ}_{u}$ in Equation (20) denotes a function of A.

(f) Computation of Mac overhead:

Equation (23) can be used to model the overhead mean of MAC protocol. Let us consider h as the average value of the back-off parameters considering the security/safety selected by vehicles. Thus, h=(A−1)2, where the back-off value is determined uniformly between A−1 and 0 periods. Average overhead is calculated as follows:

(24) $$D\left(A\right)=\frac{\left[A-1\right]\varphi /2+s_{CTS}+s_{RTS}+3s_{SIFS}+s_{SYNC}+s_{SEN}}{S_{ℐ}}\;$$

where $s_{CTS}$, $s_{RTS}$ and $s_{SIFS}$ are the time corresponding of Request to Send (RTS), Clear to Send (CTS), and Short inter-frame space (SIFS) packets; $s_{SYNC}$ is the synchronization of size packets; $s_{SEN}$ is the sensing time; $S_{ℐ}$ is the time cycle, and φ is the one back-off param of corresponding time. The D. overhead depends on the results of channel allocation. Thus, D is updated in flow diagram 2 based on the current channel assigned. Our PECA minimizes collision and maximizes system throughput, and provisioning security to S-PECA does not incur much overhead as proved in the next section experimentally.

## 4. Results

The experiments are conducted on a Windows 10 operating system, 64-bit I-5 quad-core processor with 32 GB RAM and Dedicated 4 GB NVidia CUDA GPU card. The SIMITS [39] simulator tool is used for experimental evaluation. The proposed PECA, S−PECA, and existing ENCCMA and S−ENCCMA algorithms are written in C# object-oriented programing language using Visual studio framework 4.5, 2012. The PECA; S−PECA; S−ENCCMA; and city, highway, and rural radio propagating environment model (ours) are incorporated into the SIMITS tool. Experiments are conducted to evaluate the performance of S−PECA over S−ENCCMA in terms of throughput achieved, successful packet transmission, and packet collision. The experiments are conducted considering different environments such as city, highway, and rural [49,50,51].

For simulating and modeling the CHR environmental conditions, we considered the parameters presented in [52] (Table 3). Table 4 illustrates the evaluation simulation parameters.

(a) Throughput

**Figure 7:**
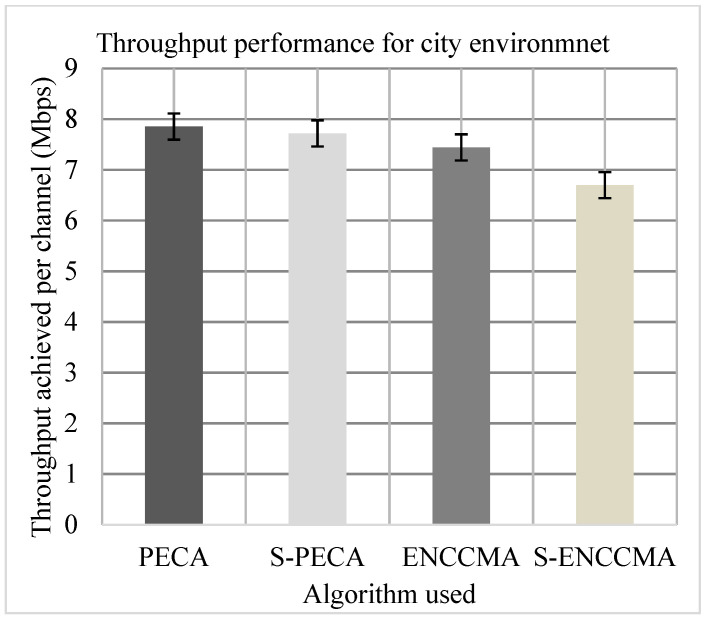
Throughput performance for the city environment.

**Figure 8:**
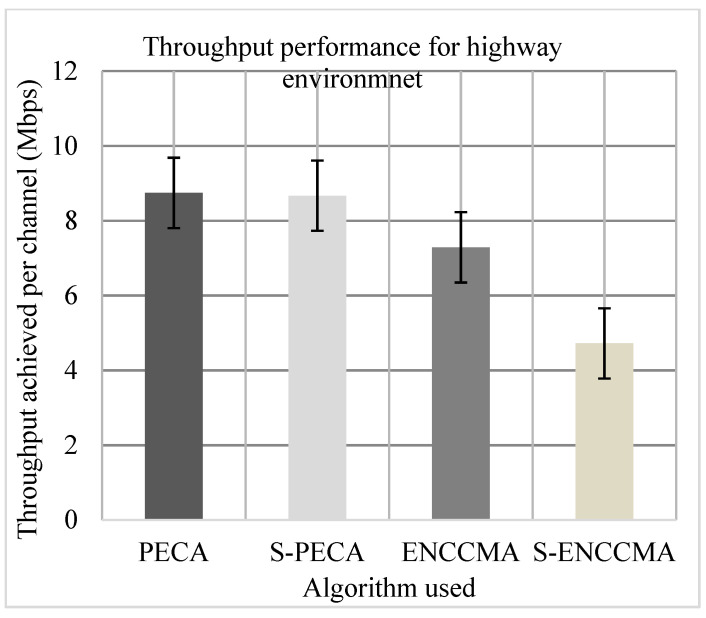
Throughput performance for highway environment.

**Figure 9:**
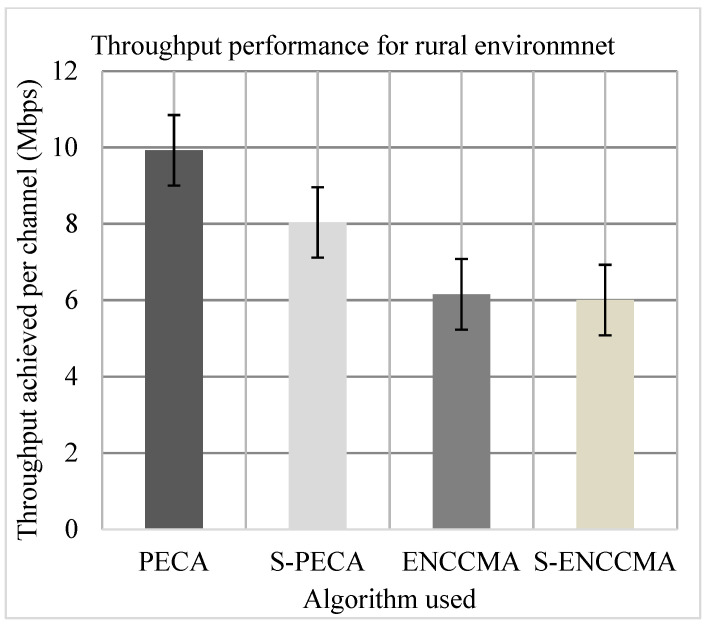
Throughput performance for the rural environment.

The experiment was evaluated to assess the productivity performance of the proposed method with the state-of-the-art mechanisms and to assess overheads for providing security/safety to VANET. Firstly, we experiment to assess the throughput of PECA and ENCCMA considering the 20 vehicles in city, highway, and rural environments indicated in Figure 7, Figure 8 and Figure 9, respectively. The experimental outcome shows that PECA improves throughput by 5.23%, 16.65%, and 37.97%, compared to ENCCMA, respectively in city, highway, and rural environments. An average throughput increased 19.95% by PECA compared to ENCCMA considering varied environmental models. Secondly, we evaluated S−PECA and S−ENCCMA by running the experiment on 20 vehicles used in city, highway, and rural environments, shown in Figure 7, Figure 8 and Figure 9, considering security scheme. The experimental outcome shows that S−PECA improves throughput by 13.22%, 45.54%, and 25.31% over S−ENCCMA in city, highway, and rural environments. Considering the different environmental models, the average throughput increase of 28.02% is improved in S−PECA compared to S−ENCCMA. The overall result shows that when a security scheme is added to PECA and ENCCMA, the model incurs an average throughput overhead of 7.2% and 15.91%, respectively, when provisioning security considering the varied environment model. Overall results show the proposed PECA model performs much better than the existing ENCCMA significantly in terms of throughput performance when provisioning security to it.

(b) Collision

**Figure 10:**
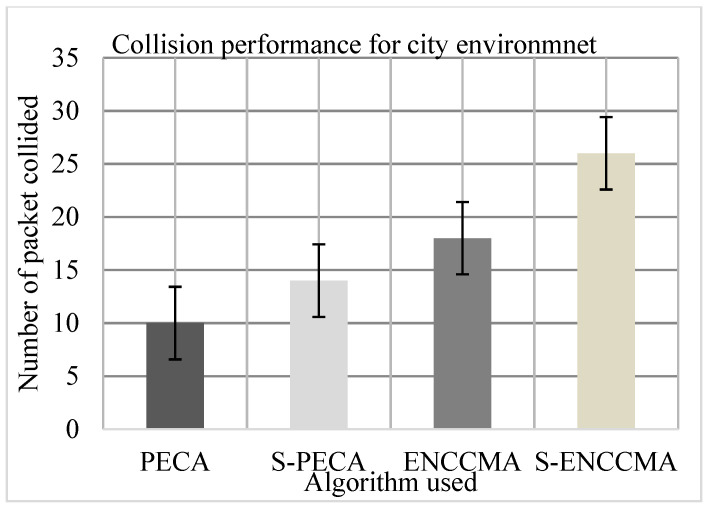
Collision performance for the city environment.

**Figure 11:**
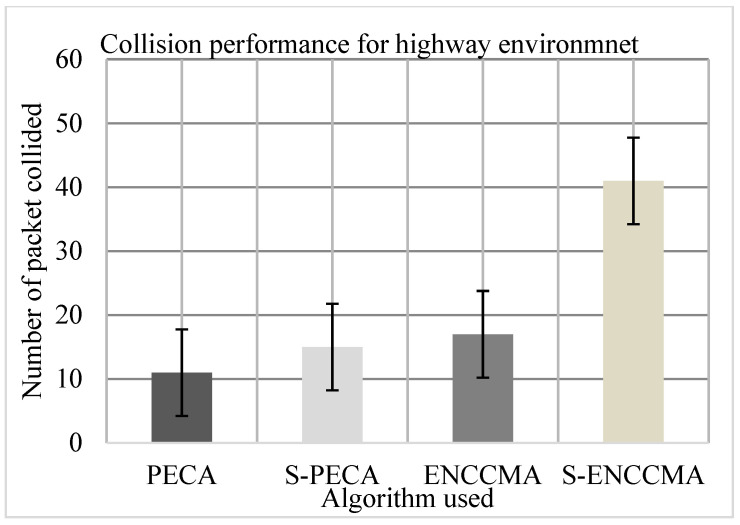
Collision performance for highway environment.

**Figure 12:**
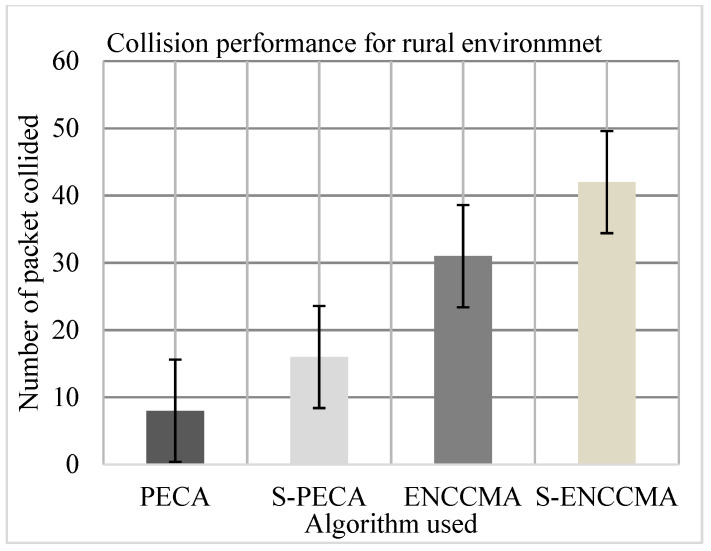
Collision performance for the rural environment.

The experiment was performed to assess the collision of the proposed method with the existing method and to assess the overhead that happened in provisioning safety to VANET. First, the experiment evaluated the collision performance of PECA and ENCCMA using 20 vehicles for the CHR environment as shown in Figure 10, Figure 11 and Figure 12, respectively. The experimental outcome shows that PECA reduces collision by 44.44%, 35.29%, and 74.13% compared to ENCCMA in city, highway, and rural environments. The average collision reduction of 51.31% is performed by PECA compared to ENCCMA considering varied environmental models. Secondly, the experiment is evaluated the collision of S−PECA and S−ENCCMA, respectively, considering 20 vehicles in the city, highway, and rural environments, shown in Figure 10, Figure 11 and Figure 12, considering security scheme. The experimental outcome shows that S−PECA reduces collision by 46.15%, 63.41%, and 61.9% over S−ENCCMA, respectively, in city, highway, and rural environments. The average collision reduction of 57.15% is performed in S−PECA compared to S−ENCCMA. considering varied environmental models. The overall result shows that when a security scheme is added to PECA and ENCCMA, the model incurs an average collision overhead of 35.07% and 15.91%, respectively, when provisioning security considering the varied environment model. The overall result obtained shows the proposed PECA model performs much better than the existing ENCCMA significantly in terms of collision performance when provisioning security to it.

(c) Performance of successful data transmission

**Figure 13:**
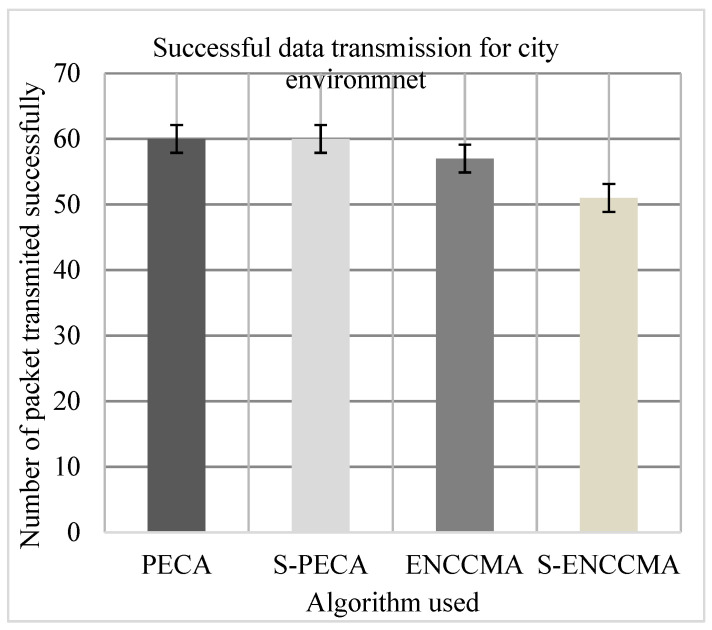
Successful transmission performance for city.

**Figure 14:**
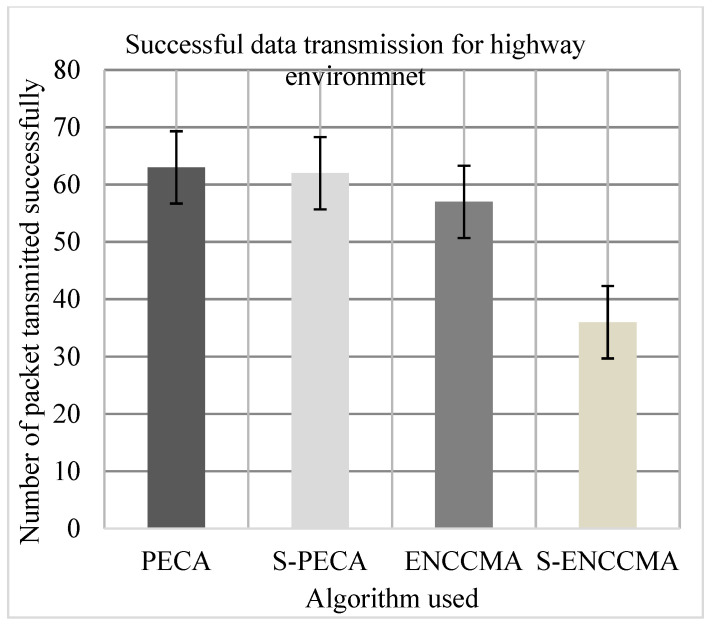
Successful transmission for highway.

**Figure 15:**
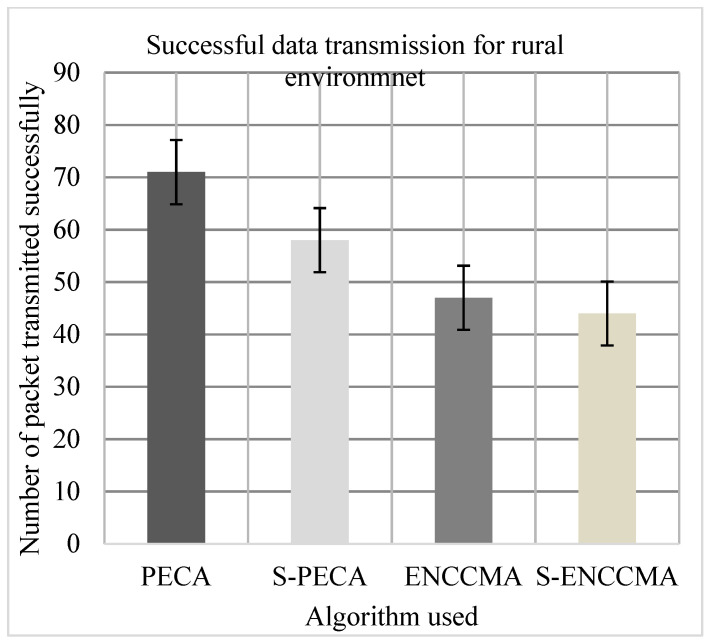
Successful transmission performance for rural.

Experiments were conducted to evaluate the packet transfer of the proposed method compared to the existing method, and the overhead of VANET security configuration was also evaluated. First, the experiment evaluated the successful transmission of PECA and ENCCMA using 20 vehicles in the various environments as we can see in Figure 13, Figure 14 and Figure 15, respectively. The experimental outcome shows that PECA performed a successful transmission of packets by 5.0%, 9.52%, and 33.8%, respectively, compared to ENCCMA in city, highway, and rural environments. The average improvement of the successful transmission is 21.66% achieved by PECA compared to ENCCMA considering varied environmental models. Secondly, the experiment was conducted to perform the packet transfer performance of S−PECA and S−ENCCMA considering 20 vehicles for C.H.R environments as shown in Figure 13, Figure 14 and Figure 15, respectively, considering security scheme. The experimental outcome shows that S−PECA improves successful packet transmission by 15.0%, 41.93%, and 24.13% over S−ENCCMA in city, highway, and rural environments. The average improvement of successful transmission is 27.02% achieved by S−PECA compared with S−ENCCMA considering varied environmental models. The overall result show that when a security scheme is added to PECA and ENCCMA, the model incurs an average successful packet transmission overhead of 6.63% and 17.97%, respectively, when provisioning security considering varied environment model. However, we can see the proposed PECA model performed much better than the existing ENCCMA, significantly in terms of successful transmission performance when provisioning security to it.

## 5. State-of-the-Art Technology Comparison

Table 5 shows the comparison between S−PECA with the state-of-the-art technology. To improve the system efficiency, S−PECA supports distribute channel sharing mechanism in V2 V environments and helps the system to achieve maximum throughput and minimum overhead. The S−ENCCMA adopts the enhanced non-cooperative cognitive division multiple access (ENCCMA) [37] real-time MAC communication protocol. To provision real-time access, the ENCCMA combines Time Division Multiple Access (TDMA), Frequency Division Multiple Access (FDMA), and Cognitive Radio (CR) techniques. The ENCCMA MAC protocol avoids signaling; this aids in enhancing the system’s efficiency. However, ENCCMA did not consider message authentication and security for personal user information. Reference [53] evaluated the performance of transmission of packet data considering different environments. However, they did not consider the movement and the numbers of the vehicles. In [54], the author performed an experimental analysis that considers different speeds for collision performance evaluation. However, their model did not consider experimental study under different environmental conditions such as city, highway, and rural and induced MAC protocol overhead [17]. Compared with the other models, our model presents a secure and efficient distributed design for channel allocation that maximizes the system throughput and reduces packet collision considering different environmental conditions. The list of the abbreviation and acronyms used in the text are presented in Table 6.

## 6. Conclusions

This work presented a secure MAC design for VANET. This model presented a commutative RSA-based channel allocation scheme on a shared channel network, namely S-PECA. The S-PECA model has overcome the key management and communication overhead issue of exiting third-party server and public-key cryptography schemes. Experiments are conducted to evaluate the overhead incurred in provisioning security to S-PECA and S-ENCCMA. The S-PECA and S-ENCCMA protocols incur an average throughput overhead of 7.2% and 15.91%, average collision overhead of 35.07% and 38.91%, and average success packet transmission overhead of 6.63% and 17.97% when security is provisioned to S-PECA and ENCCMA, respectively, considering the different environmental conditions. The outcome shows that overhead incurred by S-PECA is much lower when compared to S-ENCCMA in terms of throughput, collision, and successful packet transmission considering varied environmental models. The overall outcome shows S-PECA minimizes collision and maximizes system throughput considering different radio propagation environments when compared to state-of-the-art techniques. In future work, we would consider performance evaluation under various modulation schemes and consider designing a new security mechanism for VANET.

## Figures and Tables

**Table 1:** Communication technologies in VANET [2].

Technology	Type	Distance
2G/3G/4G/5G	Duplex	Global
Bluetooth	Simplex	≈10 m
WiMAX	Duplex	≈1 km
GPS	Simplex	global
ZigBee	Duplex	≈20 m
DSRC 802.11p/WAVE	Duplex	≈1 km
Wi-Fi	Duplex	≈50 m
RFID	Simplex	10 m

**Table 2:** Variable notation.

Notations	Abbreviation
x	Vehicle
$C_{x}$	Throughput Achieved
$e_{xy}$	Channel allocation decision
y	Channel
$V_{x}$	Channel set allocated to vehicle x
$l_{xy}$	The likelihood for channel y accessibility
$1-\underset{y\in V_{x}}{\prod }l_{xy}^{\prime }$	The likelihood for channel y accessibility for at most one channel
$l_{xy}^{\prime }$	The likelihood that channel y is not accessible
$\delta C_{x}$	Throughput increment
$V_{z}$	The input set of accessible channels
$C_{x}^{z}$	Throughput before channel allocation$\;y_{x}^{\prime }$.
$C_{x}^{q}$	Throughput after channel allocation $y_{x}^{\prime }$.
T	Is the total number of channels in the network
$l_{xy}^{\prime }=1-l_{xy}$	Is the probability of vehicle x not accessing the channel y
$y_{x}^{\prime }$	channel allocation
$1-\underset{y\in T_{x}}{\prod }l_{xy}^{\prime }$	Is the probability of x vehicle accessing the channel
D	MAC Overhead
V	Number of vehicles
T	The sharing vehicles of channel y
s	Is the common shared channel
n	The shared channel user number
m	Is the user’s number using the shared channel
$\overset{T}{\underset{m=1,m\neq n}{\prod }}l_{x_{m}y}$	Is the likelihood computation of throughput gain on a shared user channel
${ℛ}_{X}$ $\mathrm{and}\;{ℛ}_{Y}$	The region member required to securely communicate over the secure channel
j	Vehicle
$P_{j}$	A set of channels shared by j
$F_{y}$	Group of vehicles who share channel y
$P_{o}$	A set of channels shared by o vehicle
A	contention window
${ℒ}_{u}$	Likelihood of the first collision
ϵL	likelihood tradeoff
r	No. of vehicles
g	Arbitrary back-off time
${ℒ}_{u}^{\left(r\right)}$	Condition likelihood of the first collision
L{r vehicle contend}	The likelihood that r vehicles participate in the contention phase
$\wedge_{R}$	Set of all R vehicles ({1,2,3,…,R})
$\wedge_{t}$	A specific set of r user
h	Mean value of the back-off parameter
D(A)	Mean Overhead
$s_{CTS}$	Corresponding time of CTS
$s_{RTS}$	Corresponding time of RTS
$s_{SIFS}$	Corresponding time of SIFS
$s_{SYNC}$	Size of synchronization packets
$s_{SEN}$	Time of sensing
φ	A time that corresponds to one back off param
$S_{ℐ}$	Cycle Time
$A_{a}^{c}$	Prime Number
$B_{b}^{c}$	Prime Number
${ℰ}^{c}$	Public Key
$D^{c}$	Secret Key
U	Data
Y	EncData
$D_{V}$	Decryption EncData

**Table 3:** Channel parameters [52].

Environment	City	Highway	Rural
Path loss	1.61	1.85	1.79
Shadowing deviation	3.4	3.2	3.3

**Table 4:** Simulation parameters considered.

Parameters	Network	MAC	Modulation Scheme	Mobility	Bandwidth	Frequency Channels	Vehicles	Environment
Value	30 m ∗ 30 m	ENCCMA, S-ENCCMA, PECA andS-PECA	QAM-64	20 cycle per frame	27 Mbps	7	20	City, Highway, & Rural

**Table 5:** State of-art-techniques comparison.

	PECA/ S−PECA/ S−ENCCMA	ENCCMA	MS−ALOHA	SLOP	EDF−CSMA
Environment	C.H.R	Flowing vehicles freely	Highway and Urban	driver intelligent	NA
Algorithm	NSCA/SCA	(NCC− FDMA− TDMA)	MS−ALOHA	Wave−Slotted aloha	EDF−CSMA
Vehicle varied Density	Yes	No	No	No	No
Simulator used	SIMITS	SIMITS	VISSIM	YES (NA)	NS−3
MAC USED	802.11p MAC	802.11p MAC	802.11p MAC	802.11p MAC	802.11p MAC
Mobility	Yes	Yes	Yes	Yes	Yes
Channel sharing available	Yes	Yes	No	No	No
Reference	(Ours)	[37]	[53]	[54]	[17]

**Table 6:** Abbreviations and Acronyms.

Acronyms	Definition
VANET	Vehicular Ad hoc Network
S−PECA	Secure Performance Enriched Channel Allocation
S−ENCCMA	Secure Non-Cooperative Cognitive Division Multiple Access
TDMA	Time Division Multiple Access
FDMA	Frequency Division Multiple Access
RSA	Rivest–Shamir–Adleman
CR	Cognitive Radio
V2V	Vehicle to Vehicle
V2I	Vehicle to Infrastructure
V2X	Vehicle to Everything
OBU	On Board Unit
RSU	Road-Side Unit
DSRC	dedicated short range communication
MAC	Medium Access Control
MANET	Mobile Ad hoc Network
FCC	Federal Communications Commission
ITS	Intelligent Transportation Systems
RFID	Radio-frequency identification
WAVE	Wireless Access in Vehicular Environment
GPS	Global Positioning System
LTE	Long-Term Evolution
V2N	Vehicle-to-Network
PLS	Physical Layer Security
IoV	Internet of Vehicles
CRL	Certificate Revocation List
RIS	reconfigurable intelligent surface
IoT	Internet of things
SVC	Secure VANET Communication
NSCA	Non-Shared Channel Allocation
ECC	Elliptical Curve Cryptography
CRSA	Commutative RSA
CHR	City, Highway, and Rural
MS-Aloha	Mobile Slotted Aloha
VISSIM	Verkehr In Stadten Simulationsmodell
EDF−CSMA	Earliest Deadline First based Carrier Sense Multiple Access

## References

[1] Issam W., Damaj D., Serhal K., Lama A., Rached H., Zantout N., Mouftah H.T. (2021). Connected and Autonomous Electric Vehicles: Quality of Experience survey and taxonomy. Veh. Commun..

[2] Ros F.J., Ruiz P.M., Stojmenovic I. (2012). Acknowledgment-based broadcast protocol for reliable and efficient data dissemination in vehicular ad-hoc networks. IEEE Trans. Mob. Comput..

[3] Ahmed A., Rasheed H., Liyanage M. (2021). Millimeter-Wave Channel Modeling in a Vehicular Ad-Hoc Network Using Bose–Chaudhuri–Hocquenghem (BCH) Code. Electronics.

[4] Azees M., Vijayakumar P., Deborah L.J. (2016). Comprehensive survey on security services in vehicular ad-hoc networks. IET Intell. Transp. Syst..

[5] Dedicated Short Range Communications (DSRC). http://grouper.ieee.org/groups/scc32/dsrc/index.html.

[6] Keyvan A. (2021). Joint use of DSRC and C-V2X for V2X communications in the 5.9 GHz ITS band. IET Intell. Transp. Syst..

[7] Petit J., Schaub F., Feiri M., Kargl F. (2015). Pseudonym Schemes in Vehicular Networks: A Survey. IEEE Commun. Surv. Tutor..

[8] Kiela K., Barzdenas V., Jurgo M., Macaitis V., Rafanavicius J., Vasjanov A., Kladovscikov L., Navickas R. (2020). Review of V2X–IoT Standards and Frameworks for ITS Applications. Appl. Sci..

[9] Miao L., Virtusio J.J., Hua K.-L. (2021). PC5-Based Cellular-V2X Evolution and Deployment. Sensors.

[10] Mohammed A.A., Ahmed A.A., Lee H.J. V2V communication modeling for environmental channel throughput and radio propagation. Proceedings of the 8th IEEE International Conference on ICTC Convergence.

[11] Mohammed A.A., Ahmed A.A., Kang Y.J., Lee H.J. Obstacles Effects on Signal Attenuation in Line of Sight for Different Environments in V2V. Proceedings of the 20th International Conference on Advanced Communication Technology (ICACT).

[12] (2009). ITS Standards Fact Sheets. Proceedings of the IEEE 1609—Family of Standards for Wireless Access in Vehicular Environments (WAVE).

[13] Storck C.R., Duarte-Figueiredo F. (2020). A Survey of 5G Technology Evolution, Standards, and Infrastructure Associated with Vehicle-to-Everything Communications by Internet of Vehicles. IEEE Access.

[14] Mohammed A.A., Ahmed A.A., Lee H.J. Performance Analysis for City, Highway and Rural Area in Vehicle-to-Vehicle Network. Proceedings of the 8th IEEE International Conference on ICTC Convergence.

[15] Mohammed A.A., Ahmed A.A., Hind R., Lee H.J. A Novel Throughput and Collision for City Environment in V2V Communication. Proceedings of the 10th IEEE International Conference on ICTC Convergence.

[16] Mohammed A.A., Ahmed A.A., Lee H.J. Comparison between DSRC and other Short-Range Wireless Communication Technologies. Proceedings of the 2020 22nd International Conference on Advanced Communication Technology (ICACT) Phoenix Park.

[17] Chang C.Y., Yen H.C., Deng D.J. (2015). V2V QoS Guaranteed Channel Access in IEEE 802.11p VANETs. IEEE Trans. Veh. Technol..

[18] EU Road Safety Policy Framework 2021–2030—Next steps towards “Vision Zero”, European Commission, Brussels, 19.6.2019. https://ec.europa.eu/transport/sites/transport/files/legislation/swd20190283-roadsafety-vision-zero.pdf.

[19] 40+ Corporations Working on Autonomous Vehicles, 16 December 2020. https://www.cbinsights.com/research/autonomous-driverless-vehicles-corporations-list/.

[20] IEEE Connected & Autonomous Vehicles. https://site.ieee.org/connected-vehicles/news/news/.

[21] Ho T.M., Tran T.D., Nguyen T.T., Kazmi S.M.A., Le L.B., Hong C.S., Hanzo L. (2019). Next-generation wireless solutions for the smart factory, smart vehicles, the smart grid and smart cities. arXiv.

[22] Contreras-Castillo J., Zeadally S., Guerrero-Ibáñez J. (2018). Internet of Vehicles: Architecture, Protocols, and Security. IEEE Internet Things J..

[23] Bharat M., Sree K.S., Kumar T.M. (2014). Authentication solution for security attacks in VANETs. Int. J. Adv. Res. Comput. Commun. Eng..

[24] Farash M.S., Turkanović M., Kumari S., Hölbl M. (2016). An efficient user authentication and key agreement scheme for heterogeneous wireless sensor network tailored for the Internet of Things environment. Ad Hoc Netw..

[25] Li H., Lu R., Zhou L., Yang B., Shen X. (2014). An efficient Merkletree- based authentication scheme for smart grid. IEEE Syst. J..

[26] Li H., Liu D., Dai Y., Luan T.H. (2015). Engineering searchable encryption of mobile cloud networks: When QoE meets QoP. IEEE Wirel. Commun..

[27] Qu F., Wu Z., Wang F.Y., Cho W. (2015). A security and privacy review of VANETs. IEEE Trans. Intell. Transp. Syst..

[28] He D., Zeadally S., Xu B., Huang X. (2015). An efficient identity-based conditional privacy-preserving authentication scheme for vehicular adhoc networks. IEEE Trans. Inf. Forensics Secur..

[29] Kafle V.P., Fukushima Y., Fujikawa K., Harai H. (2016). ID-based communication framework in future networks. Wirel. Pers. Commun..

[30] Zhou A., Li J., Sun Q., Fan C., Lei T., Yang F. (2015). A security authentication method based on trust evaluation in VANETs. EURASIP J. Wirel. Commun. Netw..

[31] Li W., Song H. (2016). ART: An attack-resistant trust management scheme for securing vehicular ad hoc networks. IEEE Trans. Intell. Transp. Syst..

[32] Wagan A.A., Jung L.T. Security framework for low latency VANET applications. Proceedings of the IEEE International Conference on Computer and Information Sciences (ICCOINS).

[33] Suresh J.S., Jongkun L. (2015). A TPM-based architecture to secure VANET. Indian J. Sci. Technol..

[34] Rehman A., Hassan M.F.B. (2019). Design Specification of Context Cognitive Trust Evaluation Model for V2V Communication in IoV. Emerging Trends in Intelligent Computing and Informatics, (IRICT 2019). Adv. Intell. Syst. Comput..

[35] Rajput U., Abbas F., Oh H. (2016). A Hierarchical Privacy Preserving Pseudonymous Authentication Protocol for VANET. IEEE Access.

[36] Liu Y., Wang Y., Chang G. (2017). Efficient Privacy-Preserving Dual Authentication and Key Agreement Scheme for Secure V2V Communications in an IoV Paradigm. IEEE Trans. Intell. Transp. Syst..

[37] Al-Absi M.A., Al-Absi A.A., Lee H.J. (2019). Performance Enriching Channel Allocation Algorithm for Vehicle-to-Vehicle City, Highway and Rural Network. Sensors.

[38] Han Y., Ekici E., Kremo H., Altintas O. (2017). Throughput-Efficient Channel Allocation Algorithms in Multi-Channel Cognitive Vehicular Networks. IEEE Trans. Wirel. Commun..

[39] Manzano M., Espinosa F., Ángel M., Santos B., Vicente A.G. (2015). Cognitive Self-Scheduled Mechanism for Access Control in Noisy Vehicular Ad Hoc Networks. Hindawi Publishing Corporation. Math. Probl. Eng..

[40] Kasana R., Kumar S., Kaiwartya O., Yan W., Cao Y., Abdullah A. (2017). Location error resilient geographical routing for vehicular ad-hoc networks. IET Intell. Transp. Syst..

[41] Makarfi A.U., Rabie K.M., Kaiwartya O., Xingwang Li X., R. Kharel R. Physical Layer Security in Vehicular Networks with Reconfigurable Intelligent Surfaces. Proceedings of the 2020 IEEE 91st Vehicular Technology Conference (VTC2020-Spring).

[42] Hsiao H., Studer A., Chen C., Perrig A., Bai F., Bellur B. Floodingresilient Broadcast Authentication for VANET. Proceedings of the 17th Annual International Conference on Mobile Computing and Networking (MobiCom).

[43] Moayad A., Safa O., Ismaeel A.R., Yaser J. (2019). An intrusion detection system for connected vehicles in smart cities. Ad Hoc Netw..

[44] Balasubramanian V., Aloqaily M., Reisslein M. (2021). An SDN architecture for time sensitive industrial IoT. Comput. Netw..

[45] Ridhawi I.A., Otoum S., Aloqaily M., Jararweh Y., Baker T. (2020). Providing secure and reliable communication for next generation networks in smart cities. Sustain. Cities Soc..

[46] Wei Z., Yanjiang Y., Wu Y., Weng J., Deng R.H. (2017). HIBS-KSharing: Hierarchical Identity-Based Signature Key Sharing for Automotive. IEEE Access.

[47] Cui J., Zhang J., Zhong H., Xu Y. (2017). SPACF: A Secure Privacy-preserving Authentication Scheme for VANET with Cuckoo Filter. IEEE Trans. Veh. Technol..

[48] Huang H.C.C., TABF Editorial Board (2020). Basic Knowledge on FinTech.

[49] Al-Absi M.A., Al-Absi A.A., Lee H.J. (2020). Varied density of vehicles under city, highway and rural environments in V2V communication. Int. J. Sens. Netw..

[50] Mohammed A.A., Ahmed A.A., Kim T., Lee H.J. (2018). An Environmental Channel Throughput and Radio Propagation Modeling for Vehicle-to-Vehicle Communication. Int. J. Distrib. Sens. Netw..

[51] Al-Absi M.A., Al-Absi A.A., Sain M., Lee H. (2021). Moving Ad Hoc Networks—A Comparative Study. Sustainability.

[52] Bilgin B.E., Gungor V.C. (2013). Performance Comparison of IEEE 802.11p and IEEE 802.11b for Vehicle-to-Vehicle Communications in Highway, Rural, and Urban Areas. Int. J. Veh. Technol..

[53] Bazzi A., Zanella A., Masini B.M. (2015). An OFDMA-Based MAC Protocol for Next-Generation VANETs. IEEE Trans. Veh. Technol..

[54] Ferreira N.F.G.C., Fonseca J.A.G. Improving Safety Message Delivery through RSU’s Coordination in Vehicular Networks. Proceedings of the 2015 IEEE World Conference on Factory Communication Systems (WFCS).

